# 68. Comparison of Cardiovascular Risk Assessment Calculators in the US Military HIV Natural History Study

**DOI:** 10.1093/ofid/ofab466.068

**Published:** 2021-12-04

**Authors:** Andrew C Wyatt, Xiaohe Xu, Colton Daniels, Thankam Sunil, Melissa Grance, Niraja Bohidar, Caitlin G Batzlaff, Anuradha Ganesan, Brian Agan, Derek Larson, Jason Okulicz, Ana E Markelz

**Affiliations:** 1 Brooke Army Medical Center, San Antonio, Texas; 2 University of Texas at San Antonio, San Antonio, TX; 3 University of Tennessee, Knoxville, TN, USA, Knoxville, Tennessee; 4 Infectious Disease Clinical Research Program, Uniformed Services University of the Health Sciences, Bethesda, MD, USA, Bethesda, Maryland; 5 Infectious Disease Clinical Research Program and the Henry M. Jackson Foundation for the Advancement of Military Medicine and Walter Reed National Military Medical Center, Bethesda, MD; 6 Infectious Disease Clinical Research Program, USU/HJF, Bethesda, Maryland; 7 Fort Belvoir Community Hospital Infectious Disease, Fort Belvoir, Virginia; 8 Brooke Army Medical Center, JBSA Fort Sam Houston, TX, San Antonio, Texas

## Abstract

**Background:**

People living with HIV (PLHIV) have increased risk of cardiovascular disease (CVD), however CVD risk assessment can be challenging as HIV-related factors are not included in most calculators. We compared CVD risk calculators in US Military HIV Natural History Study (NHS) participants.

**Methods:**

The NHS database was screened for participants enrolled between 2009-2019 who were ≥ 40 years of age with no previous history of CVD or statin use. Of the 399 participants meeting criteria, 385 (96.5%) had available data to assess 3 CVD risk calculators: Atherosclerotic CVD risk calculator (ASCVD), Framingham Risk Calculator (FRC), and the Data Collection on Adverse Eﬀects of Anti-HIV Drugs Study (DAD) risk calculator. Risk calculators were applied cross-sectionally at the first available time point at or after age 40 years and calculators were compared using a Wilcoxon signed rank test. Demographic and HIV-related characteristics were analyzed as independent variables.

**Results:**

Participants were predominantly male (91.1%), mostly White (49.6%) or Black/African American (44.7%), and commonly had a history of tobacco use (38.9%). The mean age at HIV diagnosis and at CVD risk calculation was 33 and 41.8 years, respectively (Table 1). Overall, there was significant variability between calculators with mean scores of 3.66%, 2.50% and 1.38% for ASCVD, FRC, and DAD, respectively for all pairwise comparisons (p< 0.001; Table 2). When assessing those with CVD risk ≥ 7.5%, a clinically relevant threshold, the proportion of individuals with risk ≥ 7.5% varied for the ASCVD (10.4%), FRC (7.5%), and DAD (< 0.8%) calculators. Associations or trends toward higher CVD risk was observed among the various calculators for race/ethnicity and both age < 30 years and CD4 ≤ 350 cells/uL at HIV diagnosis (Table 2).

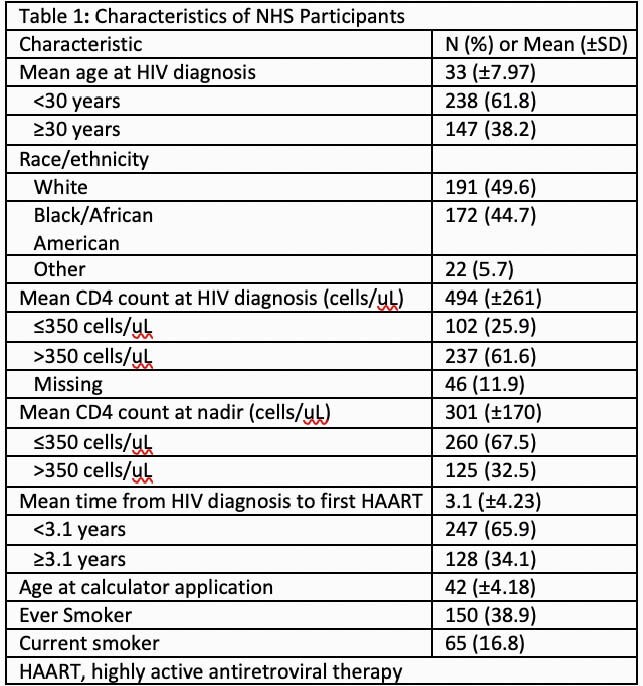

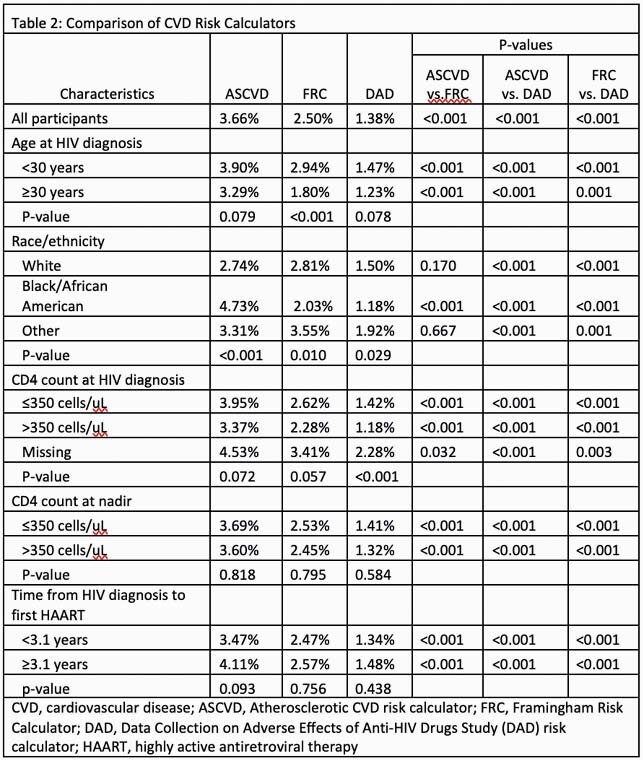

**Conclusion:**

Since significant variability among CVD risk calculators was observed in the NHS cohort, it may be challenging to apply overall CVD risk calculators in a clinically relevant manner. HIV-related factors, such as duration of HIV infection and CD4 nadir, are not accounted for in CVD calculators and may be indicators of increased CVD risk. Future studies are warranted in order to determine the optimal clinical use of CVD risk calculators for PLHIV.

**Disclosures:**

**All Authors**: No reported disclosures

